# Morphology and phylogenetic analysis of two new deep-sea species of *Chrysogorgia* (Cnidaria, Octocorallia, Chrysogorgiidae) from Kocebu Guyot (Magellan seamounts) in the Pacific Ocean

**DOI:** 10.3897/zookeys.881.34759

**Published:** 2019-10-17

**Authors:** Yu Xu, Yang Li, Zifeng Zhan, Kuidong Xu

**Affiliations:** 1 Laboratory of Marine Organism Taxonomy and Phylogeny, Institute of Oceanology, Chinese Academy of Sciences, Qingdao 266071, China Institute of Oceanology, Chinese Academy of Sciences Qingdao China; 2 Laboratory for Marine Biology and Biotechnology, Pilot National Laboratory for Marine Science and Technology (Qingdao), Qingdao 266071, China University of Chinese Academy of Sciences Beijing China; 3 Center for Ocean Mega-Science, Chinese Academy of Sciences, Qingdao 266071, China Pilot National Laboratory for Marine Science and Technology Qingdao China; 4 University of Chinese Academy of Sciences, Beijing 100049, China Center for Ocean Mega-Science, Chinese Academy of Sciences Qingdao China

**Keywords:** Anthozoa, *Chrysogorgia ramificans* sp. nov., *Chrysogorgia binata* sp. nov., gorgonian, phylogeny, taxonomy

## Abstract

Two new species of *Chrysogorgia* Duchassaing & Michelotti, 1864 collected from Kocebu Guyot in the Magellan seamounts of the Pacific Ocean are described and illustrated: *Chrysogorgia ramificans***sp. nov.** collected from a depth of 1831 m and *Chrysogorgia binata***sp. nov.** collected from a depth of 1669 m. *Chrysogorgia ramificans***sp. nov.** belongs to the *Chrysogorgia* “group A, Spiculosae” with rods distributed in body wall and tentacles, and *C. binata***sp. nov.** belongs to the “group C, Squamosae typicae” with rods and/or spindles not present but only scales. *Chrysogorgia ramificans***sp. nov.** differs from congeners by its main stem with 2/5R branching sequence at the bottom forming two large bottlebrush-shaped branches with 1/3R branching sequence at the top. *Chrysogorgia binata***sp. nov.** is similar to *C. scintillans* Bayer & Stefani, 1988, but differs by its larger polyps, larger sclerites in the body wall, and different scales in the upper part of polyps. The mtMutS genetic distances between *C. ramificans***sp. nov.** and *C. binata***sp. nov.** and congeners are in the range of 0.33%–2.28% and 0.33%–2.94%, respectively, while the intraspecific distances are in the range of 0–0.16%. Molecular phylogenetic analysis indicates that *C. ramificans***sp. nov.** is clustered with *C. monticola* Cairns, 2007 and *C. binata***sp. nov.** is clustered with *C. chryseis* Bayer & Stefani, 1988, both with high support indicating close relationships.

## Introduction

Within the gorgonian family Chrysogorgiidae, the genus *Chrysogorgia* Duchassaing & Michelotti, 1864 is the largest and most common group, distributed worldwide including the Antarctic, ranging from 100 m to 3860 m water depth. In some colonies it is characterized by a spiralling main axis that branches sympodially giving off secondary branches that subdivide dichotomously, resulting in a bottle-brush colony shape. In others, the sympodial main axis does not spiral, resulting in fan-like planar or bi-flabellate colonies (Pante and Watling 2011; Cordeiro et al. 2015). To date, *Chrysogorgia* contains 70 species (Cairns 2018). Among them, 45 species are found only from the Pacific, 17 species only from the Atlantic and 7 only from the Indian Ocean (Cairns 2001, 2007, 2018; Pante and Watling 2011; Cordeiro et al. 2015). *Chrysogorgia flexilis* Wright & Studer, 1889 occurs in both the Pacific and Indian Oceans (Wright and Studer 1889; Cairns 2001).

Based on the presence of rods or scales in the body wall and tentacles, Versluys (1902) divided *Chrysogorgia* species into three groups, which were summarized by Cairns (2001) as following: “group A, Spiculosae” (rods and/or spindles in body wall and tentacles) with 38 species, “group B, Squamosae aberrantes” (rods and/or spindles in tentacles but not in body wall) with 13 species, and “group C, Squamosae typicae” (rods and/or spindles not present; only scales) with 18 species. More recently, Cordeiro et al. (2015) described the species *C. upsilonia*, which possesses spindles in the body wall but not in the tentacles, and classified it as “group D, Spiculosae aberrantes”. The separation of four groups was further recognized by Cairns (2018).

During the investigation of the Magellan seamount benthic diversity in the tropical Western Pacific, we obtained two golden gorgonians from the Kocebu Guyot using a remotely operated vehicle (ROV). Based on morphological and phylogenetic analyses, both species proved to be new species of *Chrysogorgia* and are described as *C. ramificans* sp. nov. and *C. binata* sp. nov., respectively. Their genetic distances and phylogenetic relationships within *Chrysogorgia* are discussed.

## Materials and methods

### Specimen collection and morphological examination

**Figure 1.**
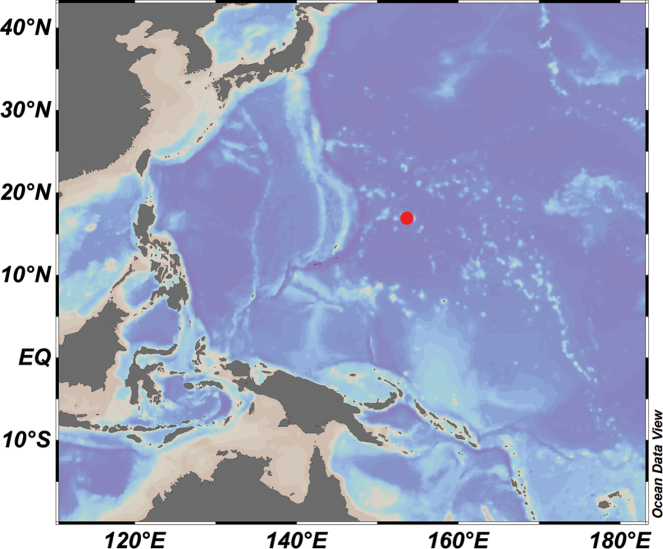
Sampling site in the Kocebu Guyot in the Western Pacific Ocean.

Specimens were obtained by the ROV*FaXian* (Discovery) from the Kocebu Guyot in the Magellan seamounts in the tropical Western Pacific during the cruises of the R/V *KeXue* (Science) in 2018 (Fig. 1). The specimens were photographed in situ before sampled, photographed onboard, and then stored in 75% ethanol after collection. Small branches were cut off and stored at -80 °C for molecular study.

The morphological terminology follows Bayer et al. (1983). A stereo dissecting microscope was used to examine the general morphology and anatomy. The sclerites of the polyps and branches were isolated by digestion of the tissues in sodium hypochlorite, and then were washed with deionized water repeatedly. To investigate the structure of polyps and sclerites, they were air-dried and mounted on carbon double adhesive tape and coated for scanning electron microscopy (SEM) observation. SEM scans were obtained and the optimum magnification was chosen for each kind of sclerites by using TM3030Plus SEM at 5 kV.

The type specimens (registration numbers: MBM286307 and MBM286346) of the two new species have been deposited in the Marine Biological Museum of Chinese Academy of Sciences (MBMCAS) at Institute of Oceanology, Chinese Academy of Sciences, Qingdao, China.

### DNA extraction and sequencing

Total genomic DNA was extracted from the polyps of each specimen using the TIANamp Marine Animal DNA Kit (Tiangen Bio. Co., Beijing, China) following the manufacturer’s instructions. PCR amplification for the mitochondrial genomic region 5’-end of the DNA mismatch repair protein – *mutS* – homolog (mtMutS) was conducted using primers AnthoCorMSH (5’-AGGAGAATTATTCTAAGTATGG-3’; Herrera et al. 2010) and Mut-3458R (5’-TSGAGCAAAAGCCACTCC-3’; Sánchez et al. 2003). PCR reactions were performed using I-5 2 × High-Fidelity Master Mix DNA polymerase (TsingKe Biotech, Beijing, China). The amplification cycle conditions were as follow: denaturation at 98 °C for 2 min, followed by 32 cycles of denaturation at 98 °C for 20 s, annealing at 50 °C for 20 s, extension at 72 °C for 15 s, and a final extension step at 72 °C for 2 min. PCR purification and sequencing were performed by TsingKe Biological Technology (TsingKe Biotech, Beijing, China).

### Genetic distance and phylogenetic analyses

All the available mtMutS sequences of *Chrysogorgia* spp. and the out-group species from related chrysogorgiidid genera were downloaded from GenBank, and those without associated publications or named *Chrysogorgia* sp. were omitted from the molecular analyses (see Table 2 and Fig. 8). The sequences were aligned using MAFFT v.7 (Katoh and Standley 2013) with the G-INS-i algorithm. Genetic distances, calculated as uncorrected “*p*” distances within each species and among species, were estimated using v.6 (Tamura et al. 2013).

For the phylogenetic analyses, only one sequence was randomly selected from the conspecific sequences without genetic divergence (see Table 2). The evolutionary model GTR+G was the best-fit model for mtMutS, selected by AIC as implemented in jModeltest2 (Darriba et al. 2012). Maximum likelihood (ML) analysis was carried out using PhyML-3.1 (Guindon et al. 2010). For the ML bootstraps, we consider values < 70% as low, 70‒94% as moderate and ≥ 95% as high following Hillis and Bull (1993). Node support came from a majority-rule consensus tree of 1000 bootstrap replicates.

Bayesian inference (BI) analysis was carried out using MrBayes v3.2.3 (Ronquist and Huelsenbeck 2003) on CIPRES Science Gateway. Posterior probability was estimated using four chains running 10,000,000 generations sampling every 1000 generations. The first 25% of sampled trees were considered burn-in trees. For the Bayesian posterior probabilities, we consider values < 0.95 as low and ≥ 0.95 as high following Alfaro et al. (2003). The accession numbers of the mtMutS sequences are listed next to the species names in the phylogenetic tree (Fig. 8).

## Results

### Class Anthozoa Ehrenberg, 1834

#### Subclass Octocorallia Haeckel, 1866

**Order Alcyonacea Lamouroux, 1812**

**Suborder Calcaxonia Grasshoff, 1999**

**Family Chrysogorgiidae Verrill, 1883**

**Genus *Chrysogorgia* Duchassaing & Michelotti, 1864**

##### Chrysogorgia ramificans sp. nov.

Taxon classificationAnimaliaAlcyonaceaChrysogorgiidae

F0FA3D58-9277-5CD3-B6F2-AC66E5601466

http://zoobank.org/DF4284E7-CC5E-4AE7-94C8-4E84366387E9

Figs 2 , 3

###### Holotype.

MBM286307, station FX-Dive 174 (17°29.93'N, 153°14.69'E), Kocebu Guyot, depth 1831 m, 8 April 2018. GenBank accession number: MK431863.

###### Diagnosis.

*Chrysogorgia* (tertiary “group A, Spiculosae” – rods or spindles in the tentacles and the body wall) with a short basal stem leading to a bottlebrush-shaped main stem, giving of a single major branch also bottlebrush-shaped. Minor branches subdivided dichotomously, up to fourth order, with the first branch internode 20–30 mm long. Branching sequence 1/3R in two large branches and 2/5R in the basal stem. Polyps 2–4 mm tall with a thin neck. Sclerites of polyp body of large and thick rods and spindles with many warts. Small scales and rods in tentacles with many warts. Scales in coenenchyme elongate with irregular edges and a few warts.

###### Description.

**Figure 2.**
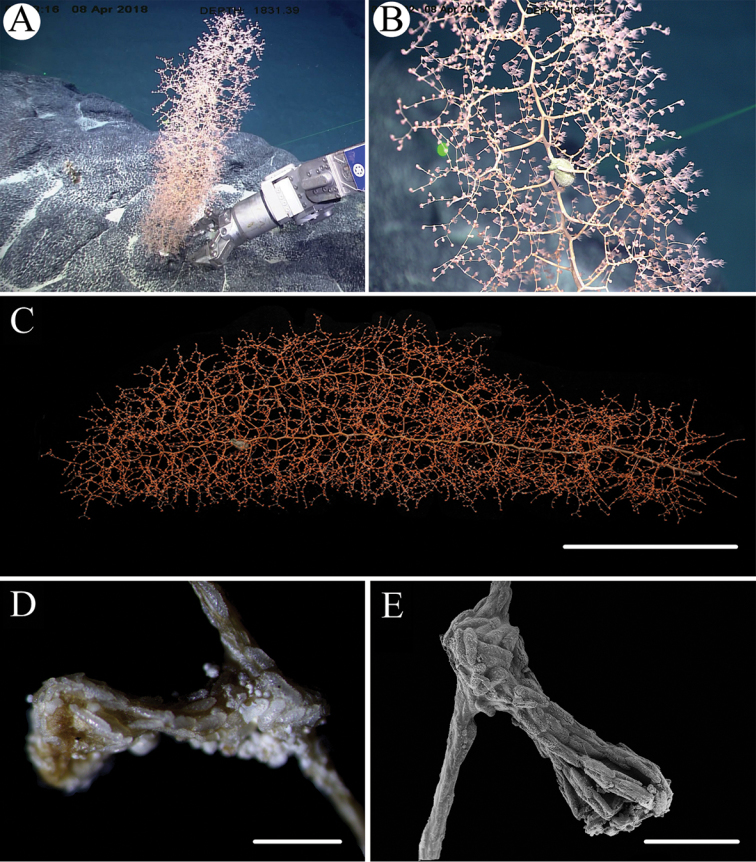
External morphology and polyps of *Chrysogorgia ramificans* sp. nov.: **A** The holotype in situ **B** Close-up of branches and polyps in situ **C** The colony after collection **D** A single polyp under light microscope **E** Single polyp under SEM. Scale bars: 20 cm (**C**); 1 mm (**D, E**).

Specimen about 73 cm long with the holdfast not recovered. Main stem forming two large bottlebrush-shaped branches whose axis has a brown metallic luster. The larger branch is 49 cm long and the other 45 cm long. The basal stem about 24 cm long and 4 mm in diameter (Fig. 2C). Branching sequences differing from bottom to top, 2/5R in the basal stem and 1/3R in the two large branches. Branches subdivided dichotomously, up to fourth order, the first branch internodes 20–30 mm long, with the terminal branchlets up to 41 mm. Distance between adjacent branches 8–12 mm, and orthostiche intervals about 30 mm in the two large branches and 50 mm at the bottom. Polyps thin, about 2–4 mm long and 1.0–1.5 mm wide at bases, with a long neck less than 1 mm wide. Two to four polyps on the first internodes, up to eight on terminal branchlets (Fig. 2D). No polyps on main axis internodes.

**Figure 3.**
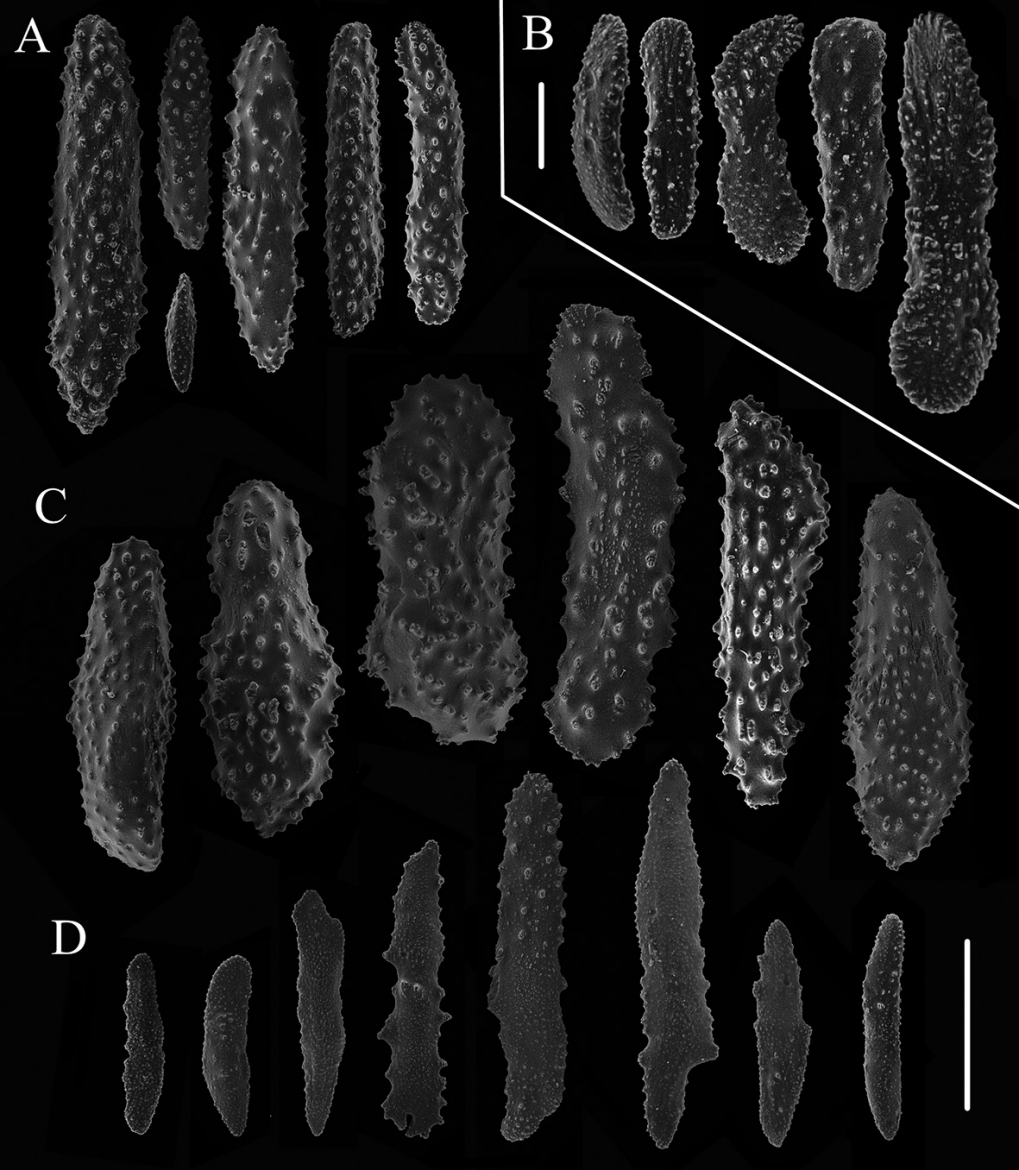
Sclerites of *Chrysogorgia ramificans* sp. nov. **A** Sclerites of polyp neck extending to the rachis of tentacles **B** Sclerites in the pinnules **C** Sclerites at the body base **D** Sclerites of coenenchyme. Scale bars: 200 μm (**A, C, D**); 50 μm (**B**).

Rods and spindles of base of polyp body wall large and thick, rarely branched, with many warts on surface, and measuring 247–628 × 109–180 μm, with an average of 430 × 136 μm (Figs 2D, 3C). Rods and spindles longitudinally arranged in the polyp neck extending to the rachis of tentacles, all covered with many warts, and measuring 95–520 × 25–96 μm, with an average of 304 × 54 μm (Fig. 3A). Scales of pinnules small with coarse edges and many warts on surface, and measuring 114–214 × 29–49 μm, with an average of 146 × 36 μm (Fig. 3B). Scales elongated and flat in coenenchyme with dentate edges and a few warts, and measuring 139–553 × 35–87 μm, with an average of 267 × 61 μm (Fig. 3D).

###### Etymology.

The Latin adjective *ramificans* (branching) refers to the ramous structure of the stem.

###### Distribution.

Found only from the Kocebu Guyot with water depth of 1831 m.

###### Habitat.

Colony attached to a rocky substrate with a small, oval-shaped holdfast (Fig. 2A).

###### Remarks.

*Chrysogorgia ramificans* sp. nov. differs from all known congeners by its main stem, with 2/5R branching sequence, forming two large bottlebrush-shaped branches with 1/3R branching sequence (Cairns 2001, 2018; Pante and Watling 2011). The new species belongs to the *Chrysogorgia* “group A, Spiculosae”, in which it mostly resembles *C. monticola* Cairns, 2007. However, *C. ramificans* sp. nov. differs from *C. monticola* by the much longer interval of adjacent branches (8–12 mm vs. 4–5 mm), longer first internode of branch (20–30 mm vs. 12 mm), larger polyps (2–4 mm vs. 1.1 mm in height), much wider rods (109–180 μm vs. 50–80 μm) with thick ends and warty surface in the body walls (vs. with thin ends and usually smooth surface), and small and unbranched rods at the tentacle base (vs. large and lobed) (Cairns 2007).

Within the group A, *Chrysogorgia ramificans* sp. nov. is also similar to *C. arborescens* Nutting, 1908, *C. tuberculata* Cordeiro et al., 2015 and *C. terasticha* Versluys, 1902. However, the new species differs from *C. arborescens* by its much longer interval of adjacent branches (8–12 mm vs. 3 mm), the higher number of polyps in the distal branchlets (up to 8 vs. 2), and usually regular sclerites (vs. irregular) (Nutting 1908). It differs from *C. tuberculata* by the larger orthostiche intervals (30–50 mm vs. 8–23 mm), rods with numerous coarse warts (vs. spindles with acute and sparse warts), rods present in tentacles (vs. only scales), and scales in coenenchyme with inconspicuous warts (vs. prominent) (Cordeiro et al. 2015). The new species can be easily distinguished from *C. terasticha* by its branching sequence (1/3R at top and 2/5R at bottom vs. 1/4L), larger orthostiche intervals (30–50 mm vs. no more than 8 mm), no nematozooids in coenenchyme (vs. many), larger polyps (2–4 mm vs. no more than 1.6 mm), larger rods with various ends (vs. smaller with rounded ends), and the absence of scales at the polyp base (vs. presence) (Versluys 1902).

##### Chrysogorgia binata sp. nov.

Taxon classificationAnimaliaAlcyonaceaChrysogorgiidae

8ED780A7-873E-52C0-B6CF-7DEDE269AA39

http://zoobank.org/D9FCB01F-49B7-4BBA-B3F0-D40026DB6519

**Figure 4.**
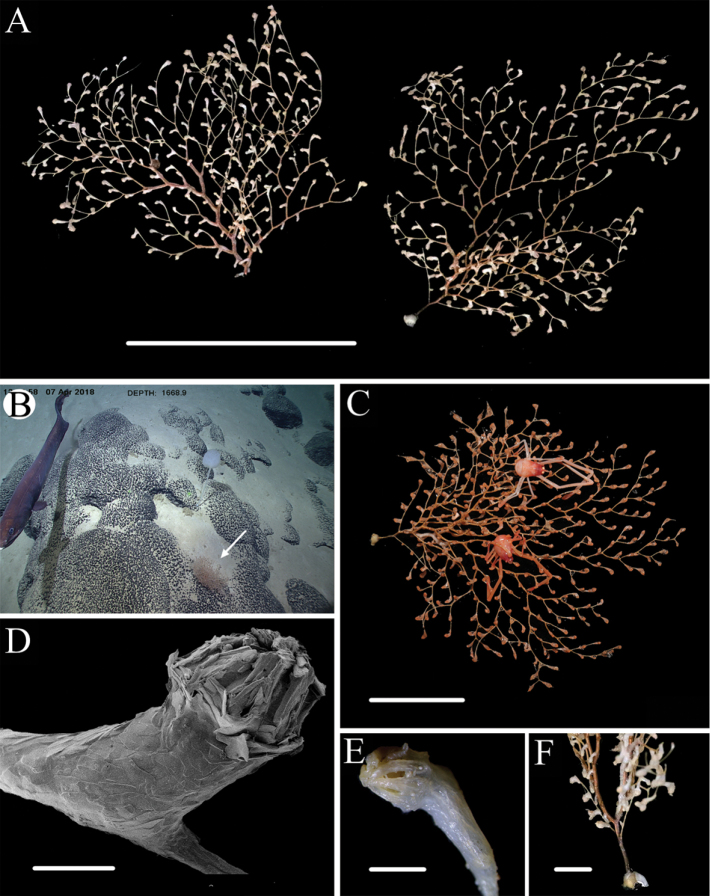
External morphology of the holotype and polyps of *Chrysogorgia binata* sp. nov. **A** Two planar fans of the colony after fixation **B** The holotype (arrow) in situ. Laser dots spaced at 33 cm used for measuring dimensions **C** The colony after collection **D** A single polyp under SEM**E** Single terminal polyp under light microscope **F** Short trunk with the first bifurcation of branches after fixation. Scale bars: 10 cm (**A**); 5 cm (**C**); 1 mm (**D, E**); 1 cm (**F**).

**Figure 5.**
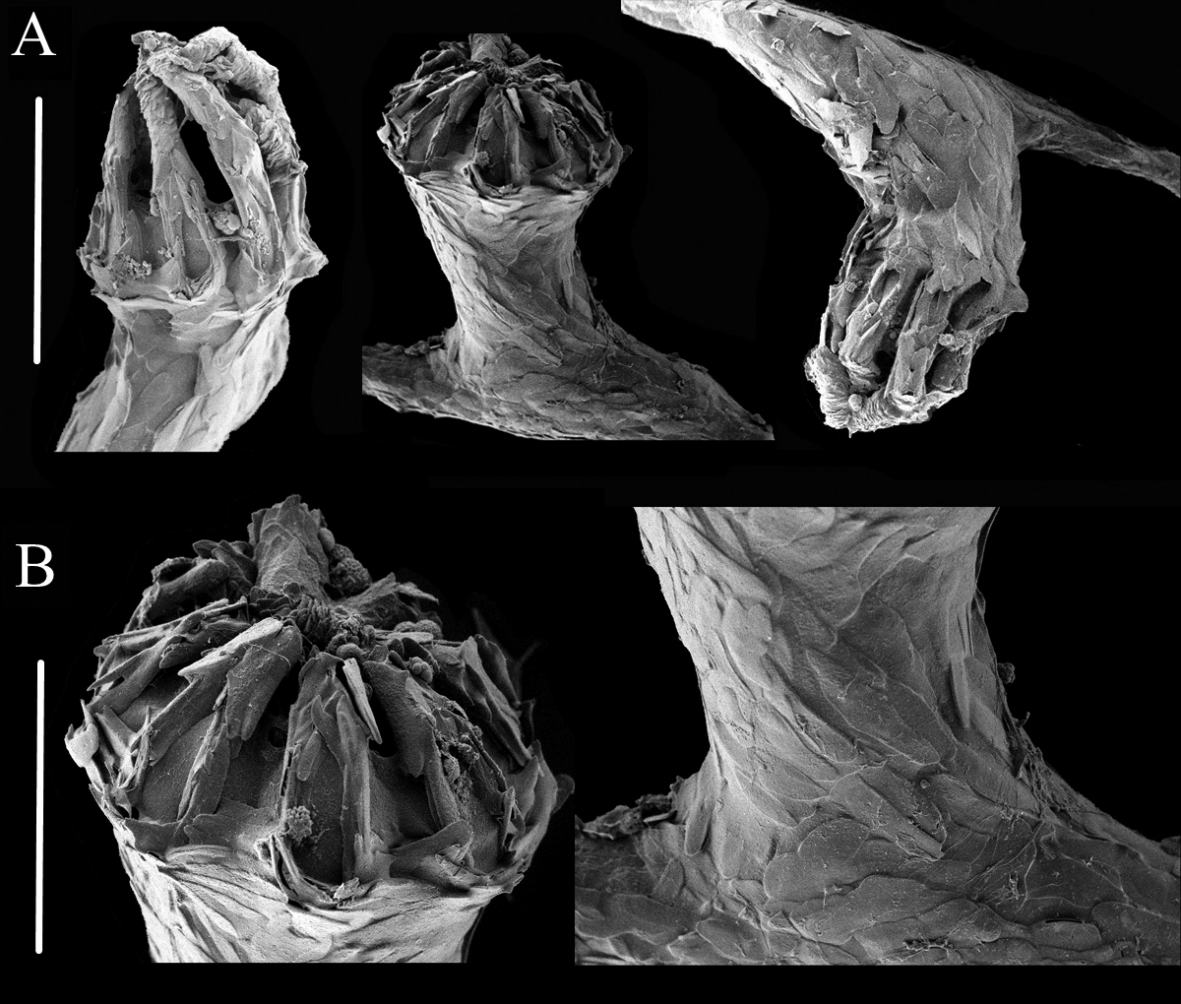
Polyps of *Chrysogorgia binata* sp. nov. **A** Three polyps under SEM**B** Head and body of one polyp under SEM. Scale bars: 2 mm (**A**); 1 mm (**B**).

**Figure 6.**
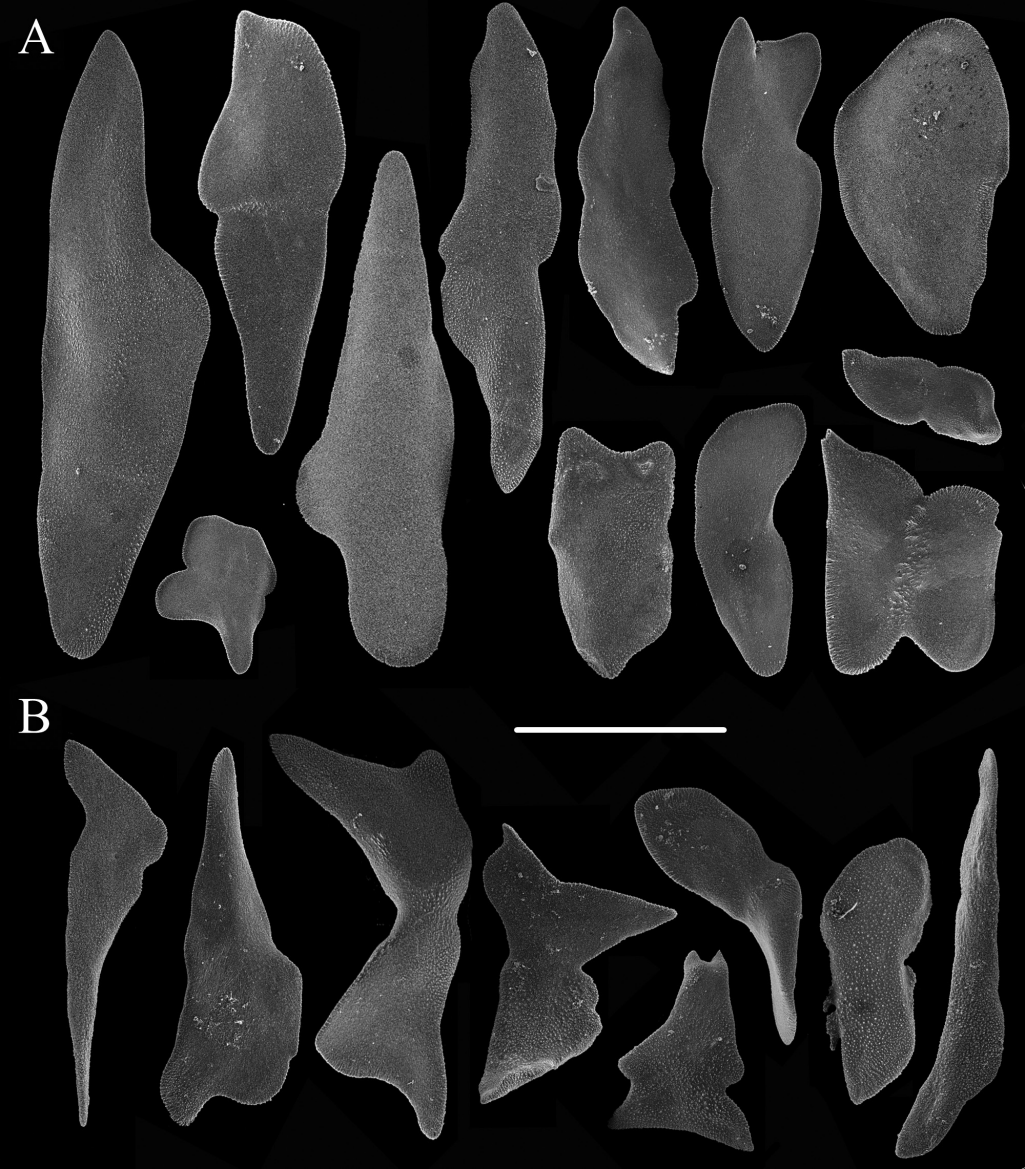
Sclerites of *Chrysogorgia binata* sp. nov. **A** Sclerites in the basal part of the polyp body **B** Sclerites in the point at the base of a naked tract below each tentacle. Scale bar: 300 μm (all at the same scale).

**Figure 7.**
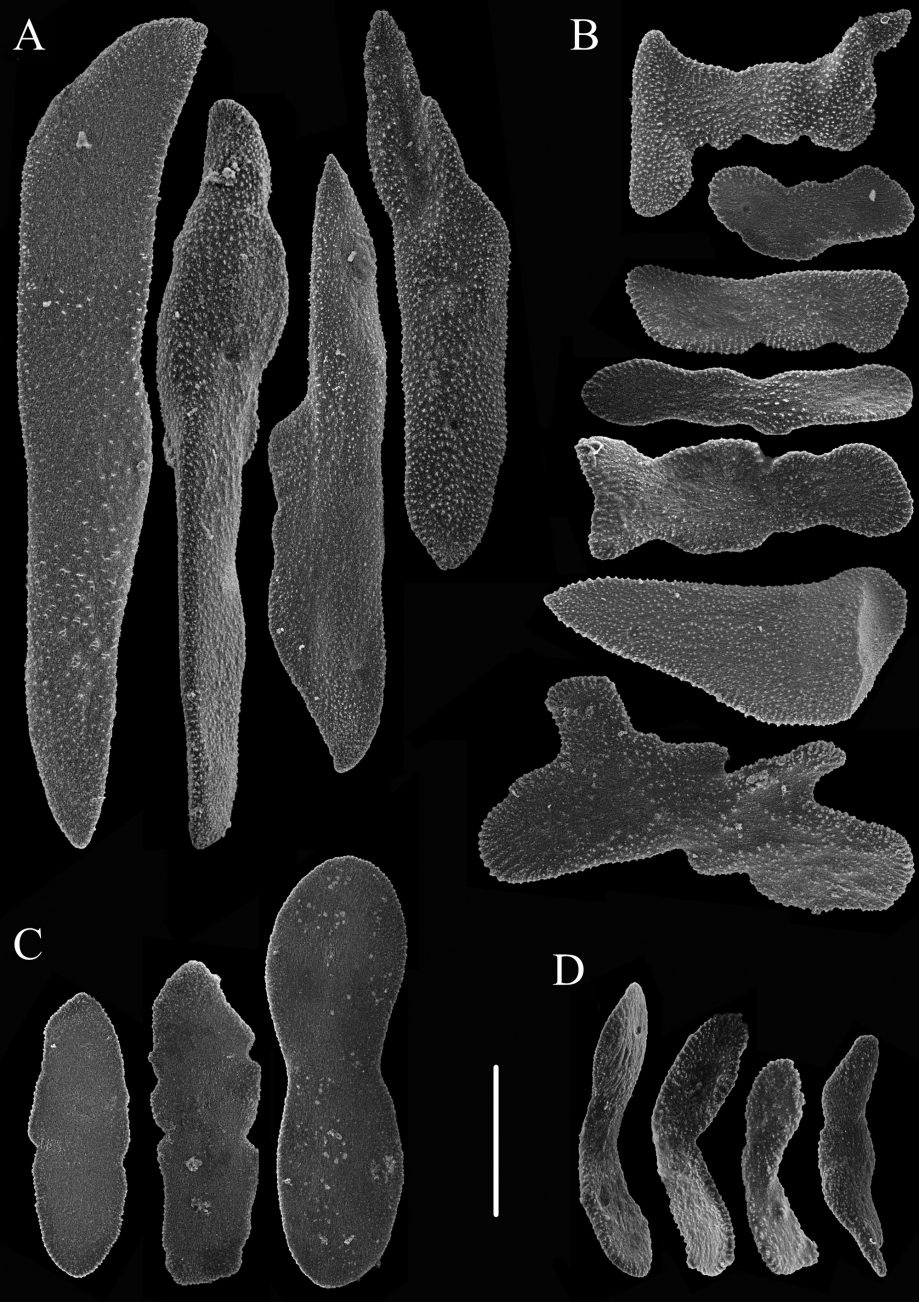
Sclerites of *Chrysogorgia binata* sp. nov. **A** Sclerites around the sides of each naked tract **B** Sclerites in the back of tentacles **C** Sclerites of the coenenchyme **D** Sclerites in pinnules. Scale bar: 100 μm (all at the same scale).

Figs 4 , 5 , 6 , 7 ; Table 1

###### Holotype.

MBM286346, station FX-Dive 173 (17°28.69'N, 153°09.95'E), Kocebu Guyot, depth 1669 m, 7 April 2018. GenBank accession number: MK431862.

###### Diagnosis.

*Chrysogorgia* (“group C, Squamosae typicae”) with a biflabellate colony and a short main stem. Polyps 3–5 mm tall. Scales smooth and thin in the basal part of polyps body with various shape, up to 1 mm long. Scales in the upper part of polyps of various shapes, converged to form an inconspicuous and blunt point at the base of a naked tract below each tentacle. Scales bluntly lancet-shaped, often with numerous coarse granules, longitudinally arranged around both sides of each naked tract. Scales irregular and coarse, usually with lobed edges in the back of tentacles. Scales of coenenchyme slipper-shaped with a medial contraction. Nematozooids absent from coenenchyme.

###### Description.

Specimen with two attached individuals of the crustacean genus *Galathea* Fabricius, 1793 (Fig. 4C). Main stem short with a principal bifurcation, forming two roughly parallel, fans (Fig. 4A, F). Calcareous holdfast small and white, about 7 mm in diameter (Fig. 4F). Major branches of each fan subdivided dichotomously or sympodially. Specimen about 16 cm long and 15 cm wide with a brilliant golden axis, and the stem about 1.5 mm in diameter at base (Fig. 4C). Internodes about 5–9 mm long, each with a single polyp except the terminal twigs, which may sometimes have two polyps. Polyps large and orange in situ, about 3–5 mm tall by 1–2 mm wide, with sclerites forming an inconspicuous blunt point at the base of a naked tract below each tentacle (Figs 4D, 5A). Terminal polyps usually with a long and narrow body (Fig. 4E).

**Table 1.** Comparison of *Chrysogorgia* species with planar structure. “–”means missing data.

Characters/Species	*C. binata* sp. nov.	*C. chryseis*	*C. desbonni*	*C. electra*	*C. pinnata*	*C. scintillans*	*C. stellata*	*C. upsilonia*
Group type	C	B	A	C	A	C	B	D
Colony shape	biflabellate	biflabellate	biflabellate	biflabellate	flabellate	biflabellate or multiflabellate	multiflabellate	flabellate
Internode length (mm)	5–9	5	3–4	6–12	3–3.5	6–7	8–10	4–30
Polyp height (mm)	3–5	up to 2	up to 2.8	1.75–2.00	up to 2.8	up to 2.75	2–4	up to 4
Eight points beneath the tentacles	short and blunt	long and sharp	inconspicuous	inconspicuous	inconspicuous	short and blunt	long and sharp	inconspicuous
Sclerites in body wall	scales various shape with low and broad marginal lobes	scales terete, tapering smoothly toward pointed ends with weak and broad marginal lobes	spindles often curved, somewhat flattened	scales elongate with narrow prominent marginal lobes	rods elongate with flattened tips	scales various shape with low and broad marginal lobes	scales terete with broad marginal lobes	spindles tuberculate
Maximum length of scale in body wall (mm)	0.93	0.7	0.75	0.6	0.56	0.65	1.1	0.67
Sclerites in tentacles	scales	rods and scales	rods and scales	scales	rods	scales	rods and scales	scales
Maximum length of rods in tentacles (mm)	0.65	0.3	0.24	–	0.21	–	0.5	0.16
Scale shape in coenenchyme	mainly slipper shape	various shape with prominent marginal lobes	elongate, warty with irregular margins	elongate, tapered with prominent marginal lobes	relatively smooth with finely serrate edges	mainly slipper shape	elongate with more or less marginal lobes	with serrate margins
Nematozooids on stem and large branches	absent	absent	–	absent	–	absent	conspicuous	–
Distribution	Western Pacific	Western Pacific	Western Atlantic	Western Pacific	Eastern Pacific	Central and Eastern Pacific	Central Pacific	South Atlantic
References	Present study	Bayer and Stefani 1988	Cairns 2001	Bayer and Stefani 1988	Cairns 2007	Bayer and Stefani 1988, Cairns 2018	Nutting 1908, Bayer and Stefani 1988	Cordeiro et al. 2015

In the basal part of the polyp body, the sclerites comprise transversally arranged, large, smooth scales. They represent a variety of shapes, a few with broad marginal lobes, length by width measuring 216–936 × 58–283 μm, with an average of 549 × 166 μm, (Figs 5B, 6A). There are also scales in the upper part of polyps of various shapes, sometimes thick and with a medial contraction, often sharp at one end , broad and lobed at the other, which combine to form an inconspicuous and blunt point at the base of a naked tract below each tentacle; measuring 275–635 × 77–254 μm, with an average of 451 × 151 μm (Figs 5B, 6B). Above these points are irregular, elongate or lancet-shaped scales mostly with coarse granules on surface, that are arranged longitudinally around the sides of each naked tract. The scales measure 337–650 × 45–85 μm with an average 431 × 70 μm (Figs 5B, 7A). The scales in the back of tentacles are coarse, of various shapes, mostly flat and lobed, and densely and transversally arranged, measuring 88–352 × 19–149 μm, with an average of 183 × 55 μm (Figs 5A, 7B). The scales in the pinnules are curved at an obtuse angle, and are sometimes thick with a few lobes on their edges; measuring 87–196 × 19–34 μm, with an average of 152 × 27 μm (Fig. 7D). The scales of the coenenchyme are generally slipper-shaped, some elongate elliptical, occasionally with indentations in their edges, and measure 138–361 × 40–87 μm, with an average of 222 × 56 μm (Fig. 7C). The coenenchyme is thin with no nematozooids.

###### Etymology.

The Latin adjective *binatus* (binate) refers to the biflabellate structure of the species.

###### Distribution.

Found only from the Kocebu Guyot in the Magellan seamounts with water depth of 1669 m.

###### Habitat.

Colony attached to a rocky substrate with a small holdfast (Fig. 4B).

###### Remarks.

Within the known species of *Chrysogorgia*, seven species mainly possess a planar structure (Table 1). Among these, including our specimen, *C. desbonni* Duchassaing & Michelotti, 1864 and *C. pinnata* Cairns, 2007 belong to the *Chrysogorgia* “group A, Spiculosae”; *Chrysogorgia chryseis* Bayer & Stefani, 1988 and *C. stellata* Nutting, 1908 belong to the “group B, Squamosae aberrantes”. *Chrysogorgia binata* sp. nov.; *C. electra* Bayer & Stefani, 1988 and *C. scintillans* Bayer & Stefani, 1988 belong to the “group C, Squamosae typicae”. The species *C. upsilonia* Cordeiro, Castro & Pérez, 2015 belongs to the “group D, Spiculosae aberrantes”. Based on the arrangement of the sclerites, *Chrysogorgia binata* sp. nov. can easily be distinguished from the species in groups A, B and D.

Both *Chrysogorgia binata* sp. nov. and *C. electra* have a biflabellate colony. However, the new species differs from the latter by its larger polyps (3–5 mm vs. generally 1.75–2.00 mm in height), eight short and blunt points beneath the tentacles (vs. inconspicuous), scales of various shapes with low and broad marginal lobes in the body wall (vs. elongate with narrow prominent marginal lobes), scales mainly slipper-shaped in coenenchyme (vs. elongate tapered with prominent marginal lobes) (Bayer and Stefani 1988). *Chrysogorgia binata* sp. nov. differs from *C. scintillans* by its larger polyps (3–5 mm vs. up to 2.8 mm in height), larger sclerites in the body wall (maximum length 0.93 mm vs. 0.65 mm), scales in the upper part of polyps (irregular and usually with sharp end vs. regular and usually with smooth end), scales around the sides of each naked tract (lancet-shaped and usually with coarse granules vs. twisted, flat and often lobed) (Bayer and Stefani 1988, Cairns 2018).

#### Genetic distance and phylogenetic analyses

Two mtMutS sequences of the two new species were obtained and deposited in GenBank, and the accession number and the length are as follows: MK431863, 695 bp for *C. ramificans* sp. nov.; and MK431862, 690 bp for *C. binata* sp. nov. The alignment datasets each comprised 649 nucleotide positions. The mtMutS genetic distances among the species of *Chrysogorgia* range from 0.16% to 2.94%, while the intraspecific distances within *C. binata* sp. nov., *C. tricaulis*, *C. artospira*, *C. averta*, *C. abludo* and *C. chryseis* are in the range 0–0.16% (Table 2). Thus, there is no distinct barcoding gap between the intra- and interspecific distances. The genetic distances between the new species *C. ramificans* sp. nov. and the known sequences of the congeners range from 0.33%–2.28%, and those between *C. binata* sp. nov. and the congeners are in the range of 0.33%–2.94% (Table 2).

**Figure 8.**
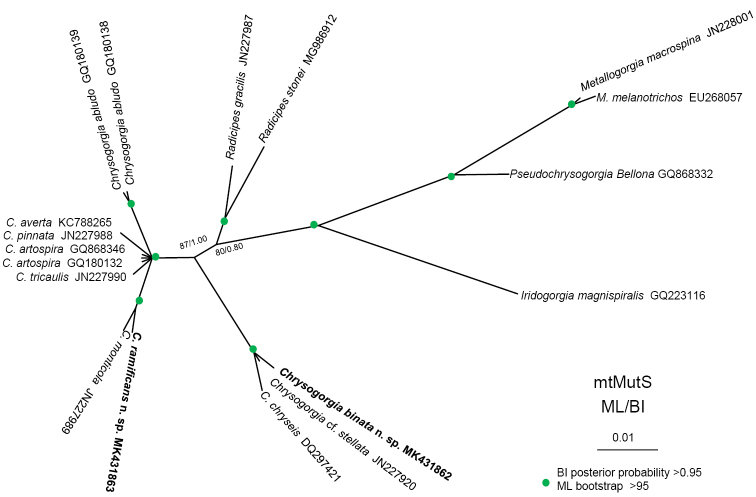
Unrooted maximum likelihood (ML) tree inferred from the mtMutS sequences of *Chrysogorgia* and related genera and species. Numbers at the nodes represent ML and Bayesian inference (BI) support values, respectively. Newly sequenced species are in bold.

The ML and BI phylogenetic trees of the mtMutS gene were nearly identical in topology and thus were combined into a consensus tree with both support values (Fig. 8). All the *Chrysogorgia* species were separated into two main groups (Fig. 8). Group I includes *C. binata* sp. nov., C. cf. stellata and *C. chryseis*, and Group II contains the subclades *C. ramificans* sp. nov. + *C. monticola*, *C. artospira*, *C. pinnata*, *C. averta*, *C. abludo*, *C. tricaulis* and *C. monticola*.

**Table 2.** Interspecific and intraspecific uncorrected pairwise distances at mtMutS of species of *Chrysogorgia* and *Radicipes*.

	Species/populations	1	2	3	4	5	6	7	8	9	10	11	12	13	14
1	***Chrysogorgia ramificans* sp. nov. MK431863**	–													
2	***C. binata* sp. nov. MK431862**	2.28%	–												
3	C. cf. stellataJN2279201	2.12%	0.16%	–											
4	*C. tricaulis*JN227990, JN227991, JN227998, GQ180123-31, EU268056	0.82%	1.79%	1.63%	0										
5	*C. artospira*GQ180132-5, GQ353317	0.65%	1.63%	1.47%	0.16%	0									
6	*C. artospira* GQ868346	0.82%	1.79%	1.63%	0.33%	0.16%	–								
7	*C. averta*KC788265, GQ180136	0.98%	1.96%	1.79%	0.49%	0.33%	0.49%	0							
8	*C. abludo*GQ180139, JN227999	1.47%	2.45%	2.28%	0.98%	0.82%	0.98%	1.14%	–						
9	*C. abludo* GQ180138	1.96%	2.94%	2.77%	1.47%	1.31%	1.47%	1.63%	0.49%	–					
10	*C. chryseis*DQ297421, JN227992	2.28%	0.49%	0.33%	1.79%	1.63%	1.79%	1.96%	2.45%	2.94%	–				
11	*C. pinnata* JN227988	0.65%	1.63%	1.47%	0.16%	0.00%	0.16%	0.33%	0.82%	1.31%	1.63%	–			
12	*C. monticola* JN227989	0.33%	2.28%	2.12%	0.82%	0.65%	0.82%	0.98%	1.47%	1.96%	2.28%	0.65%	–		
13	*Radicipes stonei* MG986912	2.28%	2.61%	2.45%	1.79%	1.63%	1.79%	1.96%	2.45%	2.94%	2.61%	1.63%	2.28%	–	
14	*Radicipes gracilis* JN227987	1.79%	2.12%	1.96%	1.31%	1.14%	1.31%	1.47%	1.96%	2.45%	2.12%	1.14%	1.79%	1.14%	–

## Discussion

*Chrysogorgia ramificans* sp. nov. mostly resembles *C. monticola* Cairns, 2007, which is also strongly supported by the phylogenetic tree and their genetic distance. However, the two species can be easily separated, as discussed above. In the phylogenetic trees, *C. binata* sp. nov., C. cf. stellata Bayer & Stefani, 1988 and *C. chryseis* Bayer & Stefani, 1988 formed a single clade with high support, indicating their close relationships (Fig. 8). However, *C. binata* sp. nov. belongs to the *Chrysogorgia* “group C, Squamosae typicae”, while *C. chryseis* belongs to “group B, Squamosae aberrantes” (Table 1; Bayer and Stefani 1988). Bayer and Stefani (1988) also reported a specimen they recorded as C. cf. stellata Nutting, 1908, which was based only on some detached branches. *Chrysogorgia binata* sp. nov. differs from C. cf. stellata by its larger polyps (3–5 mm vs. about 2 mm), many elongate or lancet-shaped scales below the tentacle base (vs. one or two rods with coarse granules), short, squarish or polygonal scales in the polyp body wall (vs. narrow and long), regular slipper-shaped scales in coenenchyme (vs. relatively irregular) (Bayer and Stefani 1988). No sequences are available for *Chrysogorgia stellata* Nutting, 1908, a species possessing a multiflabellate colony form, while *C. binata* sp. nov. is biflabellate. The new species differs from *C. stellata* also in the shorter and more blunt points beneath the tentacles (vs. long and sharp), various shapes of scales in the upper part of the body wall (vs. only a single slenderly elongate shape), and generally slipper-shaped scales in coenenchyme (vs. elongate with more lobed margin).

## Supplementary Material

XML Treatment for Chrysogorgia ramificans

XML Treatment for Chrysogorgia binata
